# Physician-staffed prehospital units for severely injured patients in the Stockholm trauma system – is there a survival benefit?

**DOI:** 10.1007/s00068-026-03270-w

**Published:** 2026-08-24

**Authors:** Kasper Helmersson, Oscar Lapidus, Tilde Alm, Denise Bäckström, Rebecka Rubensson Wahlin

**Affiliations:** 1 Department of Clinical Science, Intervention and Technology, Karolinska Institutet, Stockholm, Sweden; 2 Department of Biomedical and Clinical Sciences, Linköping University, Linköping, Sweden

**Keywords:** Trauma, Prehospital, Transport, Emergency medical services, Rapid response vehicles, Swedish trauma registry, Physician

## Abstract

**Background:**

The role of prehospital physicians for patients with major trauma has previously been examined with mixed results. No studies have assessed the survival benefit of physician-staffed prehospital units in the context of the Stockholm trauma system.

**Aim:**

To investigate the association between exposure to prehospital physicians and mortality for severely injured trauma patients in the Stockholm trauma system.

**Methods:**

A total of 11,063 trauma patients were obtained from the Swedish Trauma Registry and stratified according to physician exposure in the prehospital setting. Logistic regression was used to evaluate the association between prehospital physician exposure and 30-day mortality and Glasgow Outcome Score (GOS).

**Results:**

No significant reduction in 30-day mortality was observed in the P-EMS group compared to the EMS group (OR 1.06, *p* = 0.71). However, transport with helicopter emergency medical services (HEMS) was independently associated with greater odds of survival. The P-EMS group had significantly lower GOS at discharge compared to EMS (OR 1.72, *p* < 0.001).

**Conclusion:**

The potential benefit of prehospital physician exposure for major trauma patients remains uncertain. However, HEMS transport was associated with improved survival. Further research is needed to determine which patients benefit from prehospital physicians and to evaluate the optimal allocation of prehospital resources.

## Background

Trauma is a leading cause of disease burden worldwide, with most trauma-related deaths occurring in the prehospital phase [1, 2]. To combat this, several countries dispatch prehospital physicians in addition to emergency medical services (EMS) [3]. Prehospital physicians provide a high level of medical expertise and allow treatment and decision-making that extend beyond the scope of standard EMS protocols [4, 5]. Several studies have shown promising benefits associated with prehospital physician care in trauma such as higher triage accuracy, safer intubation, as well as access to more advanced interventions such as thoracostomy and blood transfusions [6–10]. However, studies investigating the survival benefit of physician-staffed prehospital units have yielded conflicting results [3, 10–15]. Organizational differences across prehospital systems and geographical conditions may affect the generalizability of these findings between different EMS systems [10, 16, 17]. The survival benefit of physician-led prehospital care in the context of an urban organized regional trauma system remains unclear, and this has not previously been examined in Stockholm.

## Aim

To investigate the association between exposure to prehospital physicians and mortality for severely injured trauma patients in the Stockholm trauma system.

## Methods

### Study design

This was a single-center registry-based observational study examining patients treated at the regional trauma center in Stockholm, Karolinska University Hospital in Solna. The study was reported in accordance with the Strengthening the Reporting of Observational studies in Epidemiology (STROBE) guidelines [18].

### Setting

The present study was performed in the Stockholm trauma system, which comprises 2.5 million inhabitants over a land area of 6548 km^2^, corresponding to approximately 23% of the Swedish population [19, 20]. Prehospital healthcare consists of road ambulance EMS staffed by at least one specialist nurse in prehospital care or other relevant specialty, with helicopter emergency medical services (HEMS) as well as physician-staffed rapid response vehicles available as reinforcement [21, 22]. These rapid response vehicles are staffed by an anesthesiologist or emergency physician, paired with a specialist nurse in either anesthesia or prehospital care [21]. When the data for the present study was collected, HEMS in Stockholm was staffed by nurse anesthetists, but with the possibility of rendezvous with a physician from the rapid response vehicles [23]. According to a prehospital transport directive used by EMS in Stockholm, all patients who meet predefined physiological and anatomical criteria should be transported to the designated regional trauma center, Karolinska University Hospital in Solna [24].

### Participants

The study cohort included all trauma patients ≥ 15 years old who were transported to Karolinska University Hospital in Solna between 2014 and 2023. The study exclusion criteria were secondary transfers, transport modalities other than EMS or HEMS, prehospital care provided by practitioners other than a nurse or a physician, unknown 30-day mortality and prehospital traumatic cardiac arrests. Patients were grouped according to prehospital physician exposure, i.e., physician emergency medical services (P-EMS), compared to regular EMS.

### Variables

The exposure was prehospital physician care, as registered in the ‘pre_provided’ variable in the Swedish Trauma Registry (SweTrau). The primary outcome was 30-day mortality (‘res_survival’). The secondary outcomes were the proportion of patients with Glasgow Coma Scale (GCS) ≤8 who were subject to prehospital intubation (‘pre_intubated’ and ‘pre_intub_type’) as well as favorable neurological outcome, defined as a Glasgow Outcome Score (GOS) of 4 or 5 (as compared to GOS 2–3) at the time of discharge from hospital (‘res_gos_dischg’ and ‘hosp_dischg_dest’ variables). Patients with GOS 1 (dead at the time of discharge) were not included in the analysis. The Revised Trauma Score (RTS) included in the multivariate analyses was calculated using the initial GCS, systolic blood pressure (SBP), and respiratory rate (RR) from the prehospital variables in SweTrau. In cases with missing exact values, the estimated value was used. Prehospital vital signs were stratified according to the predefined categories of the RTS and the RTS scores were calculated as originally described by Champion et al. [25].

### Data sources

SweTrau is a national trauma registry with data reported according to the Utstein Template [26, 27]. The registry includes all patients with a traumatic mechanism of injury who prompt an in-hospital trauma team activation (TTA), inpatients with a New Injury Severity Score (NISS) >15 regardless of TTA status and patients with NISS > 15 who are transferred to the hospital within seven days after the traumatic event (non-transferred patients must present to the hospital within 24 hours of the time of injury to be registered) [27]. Patients where the only injury is a chronic subdural hematoma as well as patients where a TTA is prompted without a traumatic mechanism of injury are excluded from SweTrau [27].

### Bias

Logistic regression was used to mitigate the risks of confounding by indication when assessing the survival benefits of P-EMS. A single-center design was chosen to mitigate inter-hospital treatment variability and to increase homogeneity in the examined cohort. This design comes at the cost of potential selection bias. However, due to centralized trauma care and strict transport directives, the selected cohort should in theory be representative of the regional severe trauma cohort.

### Study size

The present study included all eligible patients from the examined hospital registered in SweTrau during 2014–2023. Patients treated before 2014 were excluded due to uncertainty regarding data quality.

### Statistics

Statistical analyses were performed on the entire study cohort, with subgroup analyses for patients with NISS > 15, NISS ≥ 25, NISS ≥ 40, and for patients with GCS ≤ 8. The Shapiro-Wilk test was used to test for normal (Gaussian) variable distribution. The Mann-Whitney U test and chi-squared test were used to compare the study population demographics between the P-EMS and EMS groups. Multivariate logistic regression was used to evaluate the association between prehospital physician care and the study outcomes while adjusting for clinically relevant confounders known to predict trauma mortality. The regression model included age, sex, transport type (HEMS or EMS), injury type (blunt or penetrating), NISS, and RTS as covariates. Calibration of the regression models was assessed using the Brier score and slope. Multicollinearity was assessed using the variance inflation factor (VIF).

Sensitivity analyses were performed using inverse probability weighting (IPW) with propensity scores to assess the robustness of the mortality and GOS outcomes [28]. The probability of treatment assignment was estimated using logistic regression. Variables predicting P-EMS exposure (age, sex, transport type, injury mechanism, and NISS) were included as covariates in the model. The individual scores (0–4) of the RTS (GCS, SBP and RR) were included in the model to improve covariate balance. Stabilized weights were then calculated based on the estimated propensity score. Baseline balance between the treatment groups following IPW was assessed using standardized mean differences (SMD), with an SMD < 0.1 considered to be acceptable covariate balance. Weighted logistic regression models were used to evaluate the association between P-EMS exposure and the study outcomes, with confidence intervals calculated using robust estimator [29]. All regression models were constructed using complete case analysis. Data analysis was performed using the Python programming language. A significance level of 0.05 was used for all analyses.

## Results

A total of 11,063 patients were included in the study cohort, of which 1614 were in the P-EMS group and 9449 were in the EMS group. Patients in the P-EMS group had significantly higher NISS (17 vs 6, *p* < 0.001) compared to the EMS group (Table 1). Baseline characteristics of the study cohort before and after IPW are presented in Table 1.

**Table 1:** Baseline characteristics of the study cohort before and after IPW. Missing data: ^*a*^0% (21%), ^*b*^ 0% (21), ^*c*^10% (1053), ^*d*^10% (1122), ^*e*^1% (77), ^*f*^1% (98)

Demographics	P-EMS n = 1614	EMS n = 9449	Significance p-value	SMD	P-EMS n = 1456	EMS n = 8498	SMD
**Patient characteristics:**	**Before IPW**				**After IPW (pseudopopulation)**		
Sex (male:female), % (n)	75:25 (1216:398)	67:33 (6335:3114)	<0.001	0.184	1014:442 (70:30)	5826:2672 (69:31)	0.024
Age, median (Q1, Q3)	39 (26, 56)	41 (26, 59)	0.023	0.079	41 (27, 57)	40 (26, 59)	0.012
**Injury characteristics:**							
Blunt injury, % (n)	72% (1158)	88% (8273)	<0.001	0.400	85% (1234)	85% (7264)	0.019
Penetrating injury, % (n)	28% (456)	12% (1176)	<0.001	0.400	15% (221)	15% (1234)	0.019
ISS^*a*^, median (Q1, Q3)	11 (5, 22)	5 (1, 10)	<0.001	0.615	9 (2, 16)	5 (1, 13)	0.089
NISS^*b*^, median (Q1, Q3)	17 (6, 27)	6 (2, 17)	<0.001	0.613	10 (3, 19)	6 (3, 17)	0.047
NISS^*b*^ > 15, % (n)	52% (841)	26% (2411)	<0.001	0.567	36% (518)	29% (2487)	0.135
GCS ≤ 8^*c*^, % (n)	21% (322)	7% (585)	<0.001	0.408	11% (166)	10% (837)	0.050
**Revised Trauma Score:**							
RTS^*d*^, median (Q1, Q3)	7.84 (6.61, 7.84)	7.84 (7.84, 7.84)	<0.001	0.524	7.84 (7.11, 7.84)	7.84 (7.55, 7.84)	0.019
GCS-score^*c*^, median (Q1, Q3)	4 (3, 4)	4 (4, 4)	<0.001	0.428	4 (4, 4)	4 (4, 4)	0.040
SBP-score^*e*^, median (Q1, Q3)	4 (4, 4)	4 (4, 4)	<0.001	0.393	4 (4, 4)	4 (4, 4)	0.033
RR-score^*f*^, median (Q1, Q3)	4 (4, 4)	4 (4, 4)	<0.001	0.338	4 (4, 4)	4 (4, 4)	0.025
**Transport logistics:**							
Helicopter transport, % (n)	12% (199)	20% (1878)	<0.001	0.206	18% (256)	18% (1535)	0.012
Prehospital intubation, % (n)	15% (235)	2% (177)	<0.001	0.475	9% (125)	4% (305)	0.210

**Fig. 1:**
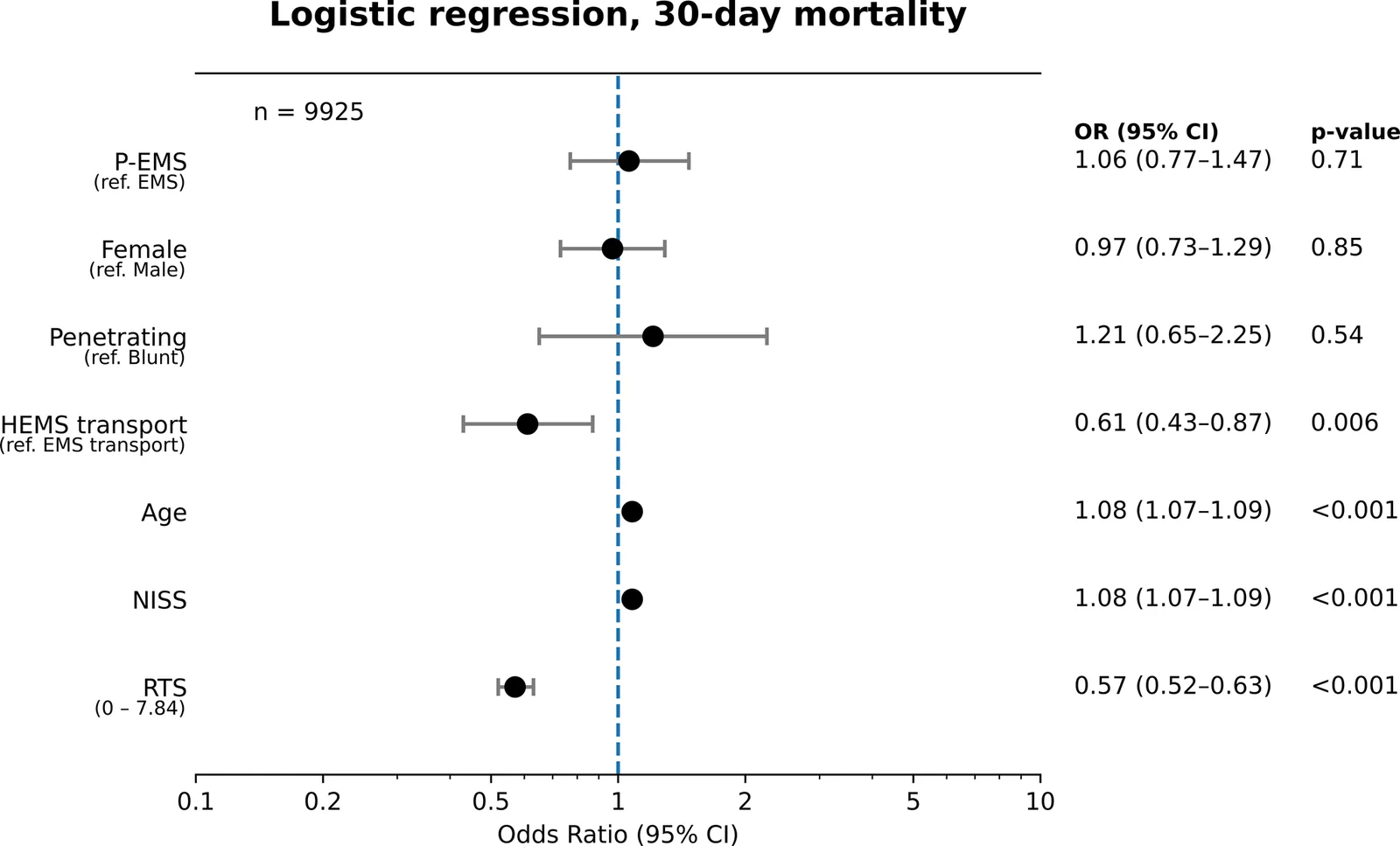
Multivariate logistic regression model for 30-day mortality. Brier score = 0.023, slope = 1.00 (95% CI = 0.93–1.07)

**Fig. 2:**
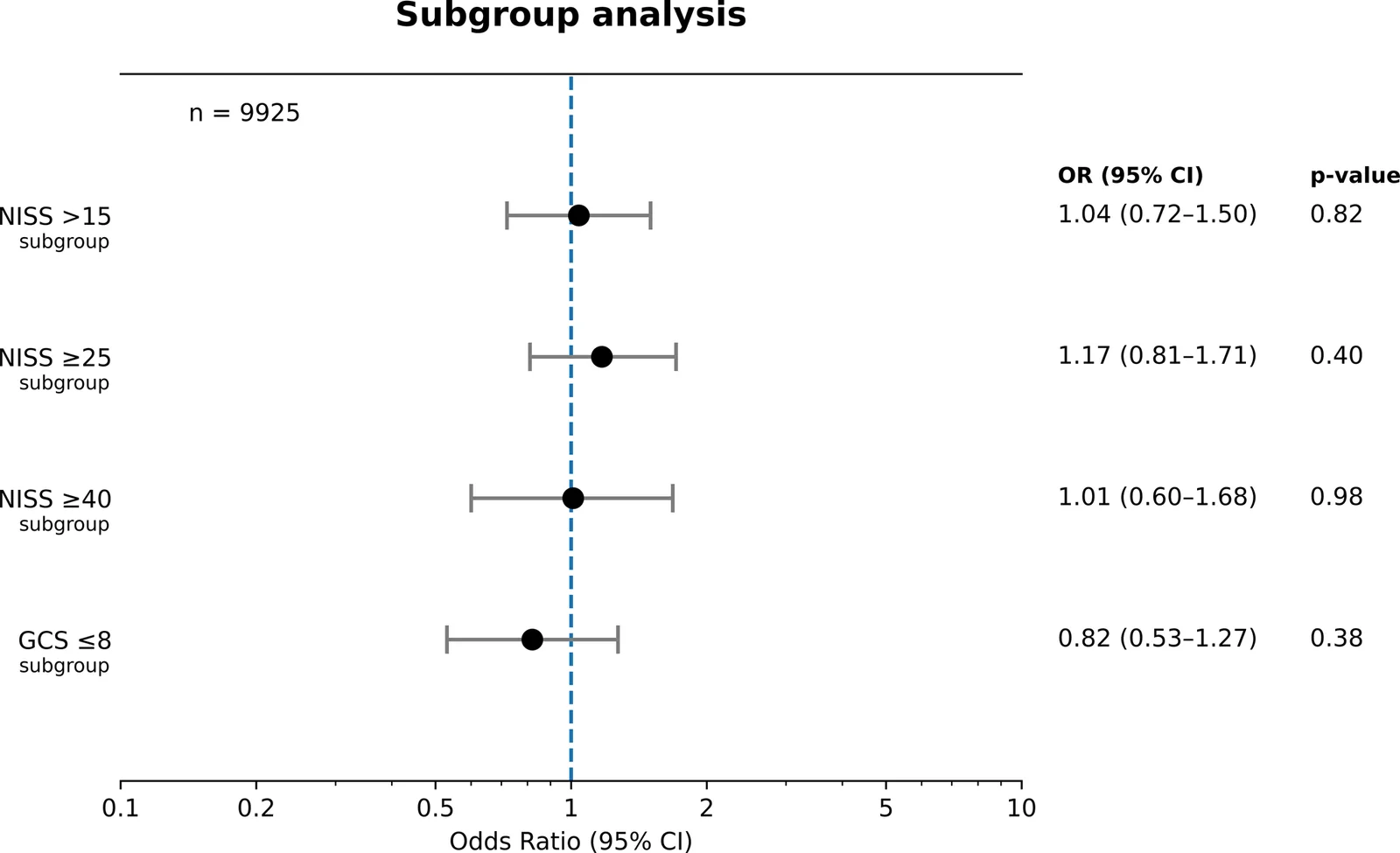
Multivariate logistic regression for 30-day mortality. Subgroup analysis is based on injury severity. The OR for P-EMS exposure is presented in the figure. EMS was used as the reference

Unadjusted 30-day mortality was significantly higher in the P-EMS group compared to the EMS group (8% vs 3%, *p* < 0.001) (Table 2). However, the multivariate logistic regression model showed no significant difference in 30-day mortality in the P-EMS group compared to the EMS group (OR 1.06, CI 0.77–1.47, *p* = 0.71) (Fig. 1). This finding was similar in the IPW sensitivity analysis (OR 0.88, CI 0.66–1.16, *p* = 0.35). Likewise, there was no significant association between P-EMS exposure and 30-day mortality in any of the examined subgroups (Fig. 2).

**Table 2:** Unadjusted outcomes for patients treated by P-EMS and EMS

Outcomes:	P-EMS	EMS	p-value
30-day mortality, % (n)	8%(129)	3%(291)	<0.001
GOS, median (Q1, Q3)	4(3, 4)	4(4, 5)	<0.001
Intubation if GCS ≤ 8, % (n)	53%(171)	22%(126)	<0.001

**Fig. 3:**
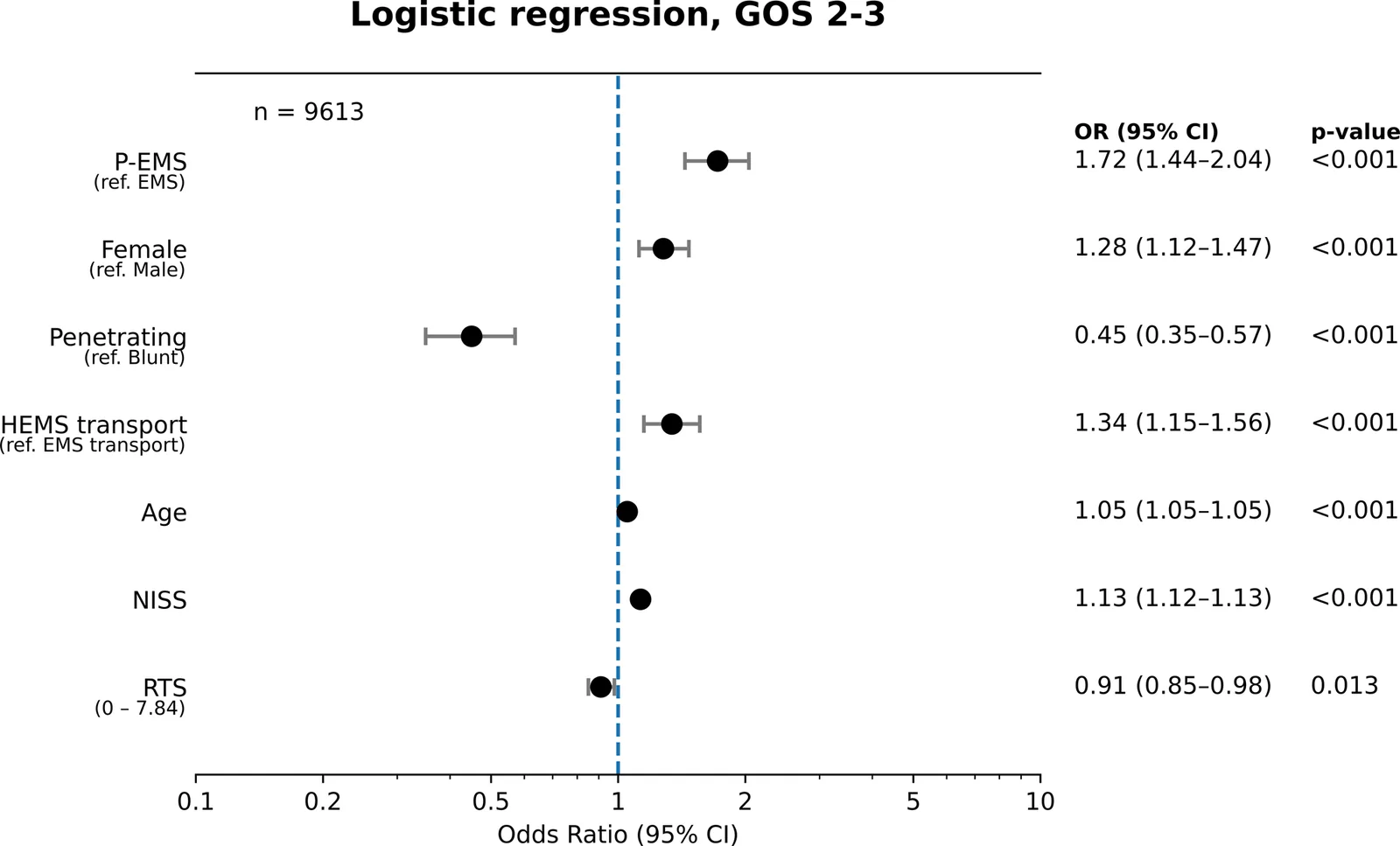
Multivariate logistic regression for unfavorable neurological outcome (GOS 2–3) at the time of discharge from the hospital. Brier score = 0.103, slope = 1.00 (95% CI = 0.96–1.04)

**Fig. 4:**
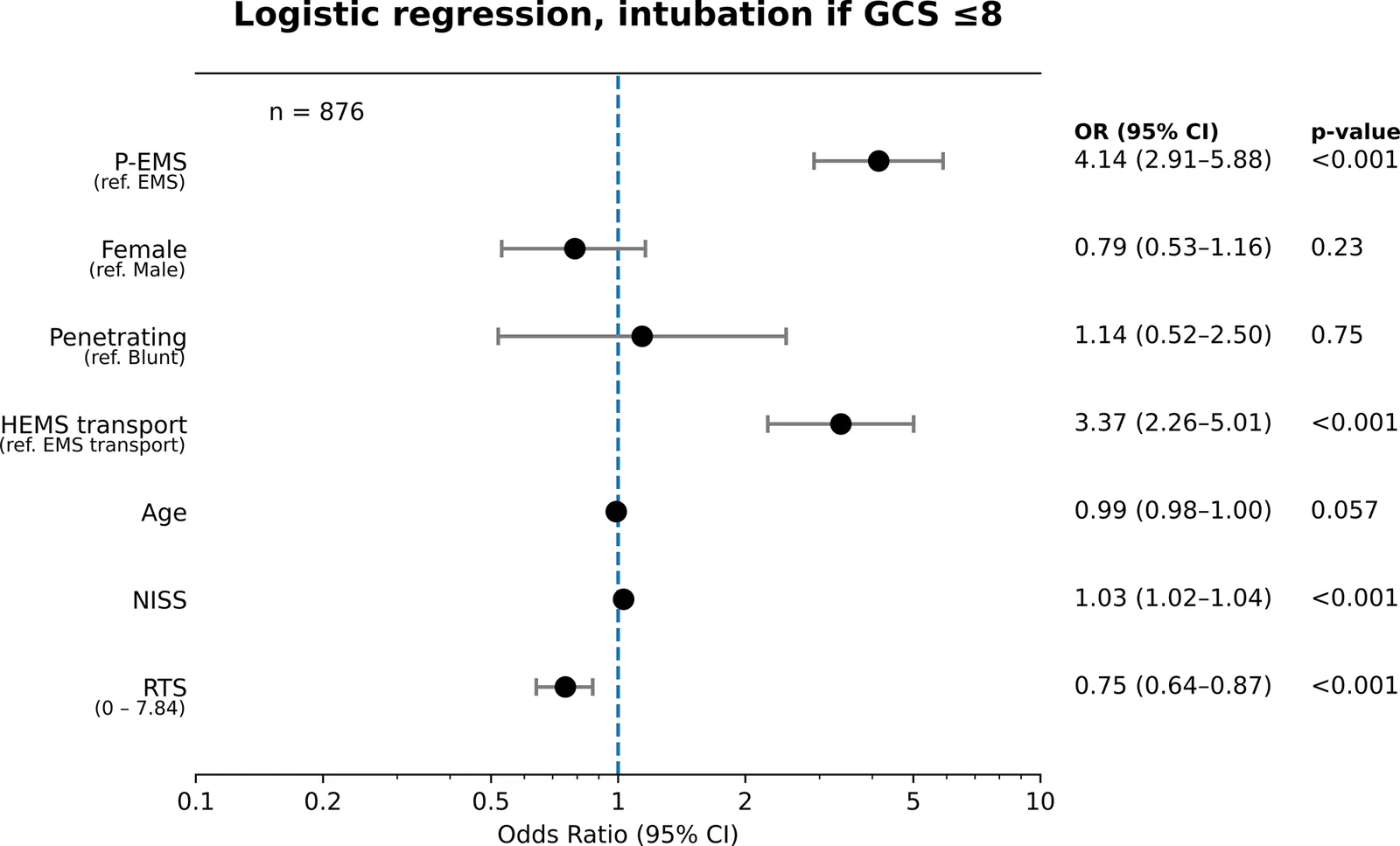
Multivariate logistic regression for intubation in patients with GCS ≤ 8. Brier score = 0.160, slope = 1.00 (95% CI = 0.85–1.15)

Patients treated by physicians had significantly lower GOS in the multivariate analysis compared to patients in the EMS group (OR 1.72, CI 1.44–2.04, *p* < 0.001) (Fig. 3). This finding was also similar in the sensitivity analysis (OR 1.60, CI 1.39–1.86, *p* < 0.001). In the subgroup of patients with GCS ≤ 8, the P-EMS group had a significantly higher intubation rate compared to the EMS group (unadjusted 53% vs 22%, *p* < 0.001), with a multivariate OR of 4.14 (CI 2.91–5.88, *p* < 0.001) after adjusting for confounding variables (Table 2, Fig. 4). Although not mutually exclusive groups, the intubation rate in patients with GCS ≤ 8 was similar between P-EMS and HEMS patients at 53% (*n* = 171) vs 55% (*n* = 107). HEMS transport was associated with higher multivariate odds of intubation, with an OR 3.37 (CI 2.26–5.01, *p* < 0.001) after adjusting for confounders (Fig. 4). The P-EMS group had significantly shorter prehospital time than the EMS group overall (Table 3). There was no substantial multicollinearity among the independent variables in any of the models (VIF < 1.42).

**Table 3:** Prehospital time intervals for patients treated by P-EMS and EMS

	P-EMS	EMS	p-value
**Prehospital time, minutes:**	*median (Q1, Q3)*	*median (Q1, Q3)*	
Total prehospital time	44 (35, 55)	50 (40, 62)	<0.001
Dispatch to arrival	9 (6, 12)	10 (7, 15)	<0.001
On-scene time	19 (12, 26)	21 (15, 29)	<0.001
Scene to hospital	15 (11, 21)	15 (11, 21)	0.684

## Discussion

The present study, which aimed to investigate the association between prehospital physician care and mortality among severely injured patients, found no significant association between P-EMS exposure and 30-day mortality. Sensitivity analysis using IPW yielded similar qualitative results, suggesting that the results were independent of the statistical method used.

This is the first study explicitly evaluating the association between prehospital physicians and trauma patient mortality in the Stockholm trauma system. Previous studies of the relationship between prehospital physicians and mortality compared to paramedic care have yielded conflicting results [3, 10–15]. However, the most recent meta-analysis reported a significant survival benefit for trauma patients treated by prehospital physicians (OR 1.39, CI 1.07–1.81) [3]. A recent nationwide study from Sweden also reported a significantly lower odds of mortality in patients treated by physicians [30]. Several factors may explain why the present study did not replicate the findings reported in previous evidence. While a common concern regarding prehospital physician involvement is the potential for prolonged prehospital times due to increased availability of advanced interventions, the present study found that P-EMS patients had significantly shorter total prehospital time and on-scene time compared to patients in the EMS group [31, 32]. The P-EMS on-scene time was also shorter than the national on-scene time reported by Lundberg et al. [30]. This suggests a prioritization of rapid transport in the P-EMS group, which may have limited physician-specific interventions to influence the outcomes. The skillset of physicians may be more valuable during longer distances with greater prehospital times, which may explain the contrasting result reported by Lundberg et al. [30]. However, as these time intervals in the present study were not adjusted for injury severity, the ability to draw definitive conclusions is limited. Furthermore, differences in staffing and training levels compared to previous studies may explain the findings in the present study. A high level of proficiency within the EMS reference group, alongside the heterogeneous composition of physician units consisting of both anesthesiologists and emergency medicine specialists could explain the non-significant finding. The proportion of anesthesiologists is higher nationwide than in the Stockholm region as prehospital physician care outside of the Stockholm region is predominantly provided by anesthesiologists [33]. This may also explain the lower odds of mortality reported by Lundberg et al. [30]. As data on physician specialty was not available in SweTrau, this could not be adjusted for in the model. Finally, despite multivariate adjustments for higher injury severity in the P-EMS group, residual confounding may persist, potentially concealing a survival benefit.

A recent Swedish study by Lapidus et al. demonstrated a survival benefit for patients transported by HEMS compared to EMS, arguing this may be due to the higher level of care provided by HEMS units [34]. Interestingly, while the present study found no survival benefit associated with exposure to a higher level of prehospital care, it did show a significant survival benefit associated with HEMS transport independent of the level of care provided in the prehospital setting.

There are several potential explanations for this finding. One possible explanation is survivorship bias, where individuals with more severe injuries could not await HEMS transport and were instead transported by regular EMS, resulting in higher mortality in the EMS group due to an inherent lower probability of survival. In the present study, attempts were made to mitigate this bias by adjusting for physiological derangement and injury severity in the multivariate analysis. It is still possible, however, that a difference in the types of missions assigned to HEMS units compared to EMS creates a demographic difference between the two groups which may account for the observed difference in mortality. Another explanation may be that helicopter transport has a true survival benefit by allowing for a more streamlined transition from the prehospital setting to the intrahospital environment. Several studies have shown that HEMS patients experience less undertriage on arrival to the hospital compared to patients transported by EMS, and the authors suggest that this may be the primary reason for improved survival in HEMS patients [34, 35].

The availability of different interventions to physicians can affect patient outcomes, which needs to be considered when comparing results between prehospital systems. Previous studies have found that prehospital intubation may be associated with improved outcome in trauma, although this is considered a high-risk intervention [36–38]. In the present study, a significantly greater proportion of patients with GCS ≤ 8 were intubated in the P-EMS group compared to the EMS group. This finding is consistent with previous literature reporting a higher rate of advanced interventions performed by physician-led teams [10, 34]. In the Swedish system, most EMS personnel do not independently provide general anesthesia without physician-staffed units present, which limits their ability to perform endotracheal intubation except for in patients with cardiac arrest. This explains the lower rate of intubation in patients with GCS ≤ 8 in the EMS group found in the present study. However, the present study also found a high OR of intubation in the HEMS group, which at this time was staffed by nurse anesthetists (this has been changed, but was the case during the study period) [23]. Interpretation of these results is complicated by the bias related to the availability of interventions highlighted previously, but the present study suggests that the survival benefit of HEMS transport was the result of factors other than intubation of patients with GCS ≤ 8, as this was seen with equal frequency in both the P-EMS and HEMS groups, with similar OR (Fig. 4).

The present study found that P-EMS patients had significantly worse neurological outcomes compared to patients treated by EMS. This finding is in line with Lapidus et al., who compared HEMS and EMS in a Swedish context and reported that patients treated by HEMS, which is often associated with a higher level of prehospital care, had worse GOS [34]. As suggested by Lapidus et al., this may be due to the nature of the injury (such as traumatic brain injury), which may be more common in groups with higher levels of prehospital care [34]. A study in Finland reported a GOS benefit in patients treated by HEMS units compared to EMS; however, this study was limited to traumatic brain injuries and patients with GCS ≤ 8 and had a small sample size [39]. The effect of a higher level of prehospital care on outcome such as GOS is likely dependent on the utilization of the time spent in the prehospital setting and the extent to which meaningful interventions are provided. As appropriate resource allocation is essential to ensure that severely injured patients have access to the highest level of prehospital care, investigations of the utilization of P-EMS and HEMS units in regional trauma systems may be warranted to ensure that they are dispatched to patients most likely to benefit from specialized care.

### Limitations

In addition to the inherent limitations of retrospective observational studies, several weaknesses need to be considered when interpreting the results of the present study. First, although the present study adjusted for multiple confounding variables, there is still a risk of inadequate adjustment and confounding by indication. In the present study, P-EMS patients had significantly lower probability of survival, higher injury severity and more penetrating injuries compared to patients in the EMS group. This was adjusted for in the multivariate analysis, but there may still be some degree of residual confounding. The present study did not adjust for injury type (other than blunt vs penetrating mechanism of injury) or patient comorbidity; however, these are also important factors which may influence survival.

Similarly to previous prehospital register studies, delayed identification of patients based on hospital admissions may introduce survival bias [5, 10]. While excluding patients with prehospital cardiac arrest further contributes to this bias, it was deemed necessary to prevent substantial confounding by indication due to marked imbalance between the exposure groups. Consequently, the findings of the present study are only applicable to patients who survived transport to the trauma center.

The present study was a single-center study focusing on the regional trauma center, which receives patients according to the prehospital trauma transport directive [24]. This design increases homogeneity and reduces bias from varying treatment effects across hospitals. Furthermore, due to limitations in registry coverage at non-trauma center hospitals in the Stockholm region, the single-center design allowed the use of high-quality data over multiple years, resulting in a larger sample size [40]. While these factors strengthen the internal validity of the study, it may come at the expense of selection bias and limiting the external validity of the results. The single-center cohort should in theory be representative of the regional severe trauma cohort due to the strict triage criteria for transport to the trauma center. However, the extent of prehospital undertriage, i.e., whether patients were transported to the appropriate hospital, is unknown. One previous study examining patients from 2008 reported that 20% of severely injured patients were initially transported to non-trauma-center hospitals in Stockholm; however, the prehospital transport directives have since been revised and are now slightly different [41]. One recent study investigating triage accuracy at a non-trauma-center hospital in Stockholm examined a relatively large cohort of severely injured patients, which could imply that the rate of prehospital undertriage is still significant; however, what percentage of the total trauma population in the region this constituted was not reported [42]. Previous studies have reported higher undertriage rates in non-physician-staffed prehospital units compared to P-EMS [6]. However, adjusting for this was deemed to be beyond the scope of this investigation.

The selection of patients who are allocated P-EMS is unknown as there are no clear criteria for dispatch. This may introduce allocation bias resulting from confounding by indication. Efforts to mitigate this bias were made by adjusting for covariates, but the extent to which this affects the results of the present study cannot be reliably estimated. A study from south-east Norway reported a P-EMS dispatch undertriage rate of 20–32% in a system lacking formalized dispatch criteria, but this has not been examined in the context of the Stockholm trauma system [43].

## Conclusion

The potential benefit of prehospital physician exposure for major trauma patients remains uncertain. However, HEMS transport was associated with improved survival. Further research is needed to determine which patients benefit from prehospital physicians and to evaluate the optimal allocation of prehospital resources.

## Data Availability

The data will not be published.

## Abbreviations

CI: Confidence interval
EMS: Emergency medical services
GCS: Glasgow Coma Scale
GOS: Glasgow Outcome Score
HEMS: Helicopter emergency medical services
ISS: Injury Severity Score
IPW: Inverse probability weighting
NISS: New Injury Severity Score
OR: Odds ratio
P-EMS: Physician-staffed emergency medical services
RR: Respiratory rate
RTS: Revised Trauma Score
SBP: Systolic blood pressure
SMD: Standardized mean difference
STROBE: Strengthening the Reporting of Observational studies in Epidemiology
SweTrau: The Swedish Trauma Registry
TTA: Trauma team activation
